# Treatment related morbidity in differentiated thyroid cancer–a survey of clinicians

**DOI:** 10.1186/1756-6614-7-3

**Published:** 2014-03-11

**Authors:** Ovie Edafe, Jonathan Wadsley, Barney J Harrison, Sabapathy P Balasubramanian

**Affiliations:** 1 Department of Oncology, University of Sheffield, Beech Hill Road, Sheffield S10 2RX, UK; 2 Department of Oncology, Weston Park Hospital, Sheffield, UK; 3 Endocrine Surgical Unit, Sheffield Teaching Hospitals NHS Foundation Trust, Sheffield, UK

**Keywords:** Thyroid cancer, Treatment, Hypocalcaemia, Voice change, TSH suppression, Morbidity, Survey

## Abstract

**Abstracts:**

**Background**

Differentiated thyroid cancer (DTC) has excellent long term survival in most patients. Long-term treatment related morbidity is therefore important, but may not be well characterised. The aim of this study was to conduct an electronic survey of clinicians involved in the care of patients with DTC to determine their views on treatment related morbidity.

**Methods**

A nine item questionnaire was developed, piloted locally and sent by email to members of three United Kingdom (UK) medical organisations (Thyroid Cancer Forum, British Association of Endocrine and Thyroid Surgeons, British Thyroid Association).

**Results**

125 responses were received. Less than 5% of respondents had a formal protocol to detect morbidity associated with TSH suppression. Over 50% agreed that morbidity from TSH suppression is not well defined. The majority of responders also agreed that the long-term morbidity of hypocalcaemia, significant voice change and TSH suppression in DTC are not well characterised. Physicians perceived treatment related morbidity to be a bigger problem than surgeons (P = 0.019).

**Conclusion**

Clinicians treating thyroid cancer agree that long-term treatment related morbidity from DTC is not well characterised. A study of the prevalence and severity of treatment related morbidity and its impact on health of patients with DTC is warranted.

## Introduction

The standard treatment regimen in most patients with differentiated thyroid cancer (DTC) includes thyroidectomy, radio-iodine (RAI) ablation and thyroid stimulating hormone (TSH) suppression therapy [1]. Diagnosis of DTC is associated with an excellent 10 and 20 year survival [2]. Long term treatment related morbidity is therefore important and includes post-thyroidectomy hypocalcaemia, the effects of TSH suppression therapy and significant voice change.

A retrospective study of 316 patients who underwent thyroidectomy for DTC found rates of transient and permanent hypocalcaemia to be 27.5% and 5.1% respectively [3]. Hypocalcaemia commonly presents with paraesthesia, muscle twitching and spasms. In more severe cases, patients may develop tetany, seizure and cardiac arrhythmias [4]. Long term hypoparathyroidism is associated with renal impairment, basal ganglia calcifications and damage to bone health [5,6].

Recurrent laryngeal nerve palsy is another potential complication following thyroid surgery and can significantly affect voice, swallowing and predispose to aspiration. Temporary and permanent recurrent laryngeal nerve palsy can occur in up to 8% and 3% of patients respectively [7]. Bilateral nerve palsy is rare and may occasionally necessitate a tracheostomy. In addition to recurrent laryngeal nerve injury, other factors affecting the quality of voice postoperatively include injury/damage to the superior laryngeal nerves, strap muscle injury and irritation or oedema of the airways [8]. Postoperative voice change after thyroidectomy can have a significant impact on an individual’s personal and professional life [9].

Although traditional practice was to continue lifelong TSH suppression treatment for all patients with thyroid cancer, recent guidelines from the American Thyroid Association (ATA) recommended that TSH suppression to less than 0.1 mU/l for initial management for DTC in high risk patients and a lower degree of TSH suppression (to between 0.1-0.5 mU/l) for low risk patients [10]. The guideline also recommended the TSH level be suppressed long term in patients with persistent disease. It is also suggested that TSH be kept at the lower limit of normal for 5-10 years in patients who were high risk at presentation and subsequently show no residual disease. Recent European guidelines suggest that patients who are free of thyroid cancer following treatment may be shifted from suppression to replacement, regardless of their initial risk [1]. There are no recommendations regarding TSH suppression in DTC patients with pre-existing bone and cardiovascular disease. Here, clinicians must balance risk from thyroid cancer recurrence and risk of bone and cardiac morbidity from TSH suppression [11].

A prospective study found that TSH suppression following thyroidectomy for PTC in women aged 50 or over was associated with a significant reduction in bone mineral density (BMD) one year after surgery [12]. In a multicentre prospective study, Bauer et al. evaluated the associated between low TSH and fracture risk in women aged 65 or over. They found that a suppressed TSH level (0.1 mU/L) increased the risk of hip and vertebrae fracture by threefold and fourfold respectively in multivariable analysis [13]. A systematic review evaluating the effects on bone metabolism of long term TSH suppression found postmenopausal women to be at increased risk of low BMD [14]. The authors recommended measurements of BMD in postmenopausal women with DTC starting TSH suppression therapy. A prospective study examined BMD in 26 pre-menopausal women having long term TSH suppression therapy for DTC [15]. They found significant reduction in BMD of femoral neck, femoral trochanter and Ward’s triangle at one-year and four-years following treatment.

The long term effect of TSH suppression on BMD, including low impact fracture rate in males has also been studied [16]. Authors found no difference in BMD or fracture rates between men on long term TSH suppressive therapy (mean ± SD duration of treatment; 15 ± 5 years) and controls. However, the study group was limited by a small sample size of 33; large sample size is required to adequately study the effect on TSH suppression on fracture rates as frequency of fractures are low in the population [16].

Adverse cardiovascular effects and symptoms associated with long term TSH suppression for DTC have been reported by various studies [17-19]. Patients receiving TSH suppression scored higher for symptoms mimicking increased adrenergic activity including palpitations and increased heart rate compared to controls [17]. One study found a significant reduction in exercise tolerance in patients with long term TSH suppression for DTC compared to matched controls [18]. This study also found significant changes in cardiac morphology including greater intra-ventricular thickness and left ventricular posterior wall thickness in patients with long term TSH suppression for DTC compared to controls. An increase in ventricular mass following long term TSH suppression for DTC was also confirmed by another group [19].

### Aims and objectives

This project aims to understand perceptions of clinicians involved in the management of DTC on long term treatment related morbidity. The objectives were to conduct an internet based survey of clinicians involved in the care of patients with DTC to understand their perceptions of treatment related morbidity, with particular reference to hypocalcaemia, significant voice change and TSH suppression.

## Methods

A questionnaire survey incorporating nine items was designed to address information pertinent to the objectives outlined above. A web based tool (http://www.surveymonkey.com) was used to create the questionnaire. Responses to treatment related morbidities were recorded on a Likert scale.

A pilot version was sent via email (embedded link to the web survey) to consultants involved in the care of patients with DTC. This was done to get feedback on the structure, appropriateness for purpose, design and usability of the web survey. The survey was amended in accordance with feedback received from three consultants.

All nine questions were included on one web page. Responders were able to change their answers during the survey; however, once submitted they were unable to review or change their answers. The survey was sent via email to doctors involved in the treatment of patients with thyroid cancer. These doctors were approached via national organisations in the UK whose members were doctors likely to be involved in thyroid cancer management. An email explaining the study and a link to the online survey was then forwarded by the secretaries of the organisations to their members.

The following UK organisations were contacted: British Association of Thyroid and Endocrine Surgeons (BAETS), Thyroid Cancer Forum (TCF) and the British Thyroid Association (BTA) with approximately 200, 230 and 150 members respectively. Considerable membership overlap exists between the organisations. A reminder email was sent 7-12 weeks after initial contact to all organisations. The survey remained open for at least four weeks after the second reminder.

A combination of IP address, response date and time was used to detect multiple entries being recorded on the electronic survey. In cases where the questionnaires were terminated early, completed questions were included in data analysis. Any unanswered questions/missing values were excluded from data analysis. All responses were downloaded and exported directly into Microsoft Excel 2007 with a Survey Monkey professional account. This avoided errors associated with manual data entry.

### Data analysis

Percentages were used to summarise responses. The outcome measure was on a Likert scale (of 4 to 6 points). The Mann-Whitney U test was used to calculate differences in perception of treatment related morbidity between surgeons and physicians (oncologist, endocrinologist and nuclear medicine physician) and between clinicians seeing higher (>12 patients per month with DTC) and lower (≤12 patients per month with DTC) volumes of patients. The significance level was set at 0.05.

### Ethical consideration

Ethics approval was obtained from the University of Sheffield Medical School Ethics Committee and the study was conducted in accordance to the submitted protocol [SMBRER244]. An amendment was obtained to send an email reminder to all potential participants.

## Results

A total of 77 responses were received following initial contact by email. The total number of responses increased to 125 following the second reminder. Four responders were excluded from analysis as they were from outside the UK. A further three were also excluded from analysis as they were not involved in the management of patients with DTC. A total of 118 responses were analysed.

A response rate was not calculated as significant overlap exists between memberships of the organisation and collection of details that may identify members was not allowed. All responders were assumed to be consultants or senior trainees.

### Background of the respondents

The responders included 80 surgeons (67.8%), 19 oncologists (16.1%), 16 endocrinologists (13.6%) and 3 nuclear medicine physicians (2.5%). The approximate number of patients (new and follow up) with DTC (at any stage of treatment) seen each month by responders was as follow: less than one, 11 (9.3%); between one and six, 52 (44.1%); between six and twelve, (17.8%) and more than twelve, 34 (28.8%). Eighty four of 118 (72.2%) and 34/118 (28.8%) of the responders were considered to be seeing lower and higher volumes of patients respectively.

Table 1 shows the number/frequency of patients seen with treatment related morbidity by responders. Table 2 shows responses to the question: ‘Do you have a locally agreed protocol for the detection of the following problems following treatment for DTC? The proportion of clinicians who had a formal protocol for detecting temporary hypocalcaemia, long term hypocalcaemia, significant voice change, symptoms/poor quality of life from TSH suppression, and bone and cardiovascular complication of TSH suppression were 65.3%, 43.6%, 28.0%, 2.5% and 3.4% respectively (Table 2).

**Table 1:** Do you see patients with the following problems following treatment for DTC?

**Complication (number of responders)**	**Never/NA**	**Rarely (≤1/year)**	**Occasionally (≤1/month)**	**Commonly (≤6/month)**	**Frequently (>6/month)**
Temporary hypocalcaemia (118)	9 (7.6%)	18 (15.3%)	63 (53.4%)	28 (23.7%)	0 (0.0%)
Long term hypocalcaemia (118)	10 (8.5%)	51 (43.2%)	43 (36.4%)	14 (11.9%)	0 (0.0%)
Significant voice change (117)	6 (5.1%)	70 (59.8%)	36 (30.8%)	5 (4.3%)	0 (0.0%)
Symptoms/poor quality of life from TSH suppression (118)	19 (16.1%)	57 (48.3%)	30 (25.4%)	12 (10.2%)	0 (0.0%)
Bone/CVS complications from TSH suppression (118)	41 (34.7%)	58 (49.2%)	19 (16.1%)	0 (0.0%)	0 (0.0%)

NA, not applicable; CVS, cardiovascular; TSH, thyroid stimulating hormone.

**Table 2:** Do you have a locally agreed protocol for the detection of the following problems following treatment for DTC?

**Complication (number of responders)**	**NA/Not involved**	**Don’t know**	**No agreed arrangements**	**Informal arrangements**	**Formal protocol**
Temporary hypocalcaemia (118)	1 (0.8%)	1 (0.8)	7(5.9%)	32 (21.7%)	77 (65.3%)
Long term hypocalcaemia (117)	2 (1.7%)	0 (0.0%)	13 (11.1%)	51 (43.6%)	51 (43.6%)
Significant voice change (118)	2 (1.7%)	2 (1.7%)	20 (16.9%)	61 (51.7%)	33 (28.0%)
Symptoms/poor quality of life from TSH suppression (118)	14 (11.9%)	5 (4.2%)	43 (36.4%)	53 (44.9%)	3 (2.5%)
Bone/CVS complications from TSH suppression (118)	17 (14.4%)	8 (6.8%)	47 (39.8%)	42 (35.6%)	4(3.4%)

NA, not applicable; CVS, cardiovascular; TSH, thyroid stimulating hormone.

Table 3 shows clinicians’ perceptions of the clinical significance of treatment related morbidity in DTC. Physicians perceived treatment related morbidity to be a bigger problem than surgeons (P = 0.019). Table 4 shows clinicians’ perceptions on how well the incidence of treatment related morbidity is defined. Clinicians seeing higher volumes of patients with DTC were more likely to agree that the incidence of the following complications is not well defined: temporary hypocalcaemia (P = 0.014), long term hypocalcaemia (P = 0.042) and significant voice change (P = 0.028). Table 5 shows clinicians’ perceptions of the characterisation of long term treatment related morbidity in DTC. Most clinicians agreed that the characterisation of long term treatment related morbidity of the following are not well defined: hypocalcaemia (55.1%), significant voice change (50.5%) and TSH suppression (57.7%).

**Table 3:** Treatment related morbidity is a significant clinical problem in patients with DTC

**Profession**	**NA**	**Strongly disagree**	**Disagree**	**Neutral**	**Agree**	**Strongly agree**
Surgeons	0 (0.0%)	4 (5.0%)	40 (50.0%)	15 (18.8%)	17 (21.2%)	4 (5.0%)
Physicians	0 (0.0%)	0 (0.0%)	12 (31.6%	10 (26.3%)	14 (36.8%)	2 (5.3%)

NA, not applicable.

**Table 4:** The incidence/prevalence of the following problems are not well defined in patients undergoing treatment for DTC

**Complication (number of responders)**	**Strongly agree**	**Disagree**	**Neutral/don’t know**	**Agree**	**Strongly agree**
Temporary hypocalcaemia (118)	16 (13.6%)	47 (39.8%)	8 (6.8%)	42 (35.6%)	5 (4.2%)
Long term hypocalcaemia (118)	16 (13.6%)	50 (42.4%)	9 (7.6%)	37 (31.4%)	6 (5.1%)
Significant voice change (117)	14 (12.0%)	42 (35.9%)	12 (10.3%)	42 (35.9%)	7 (6.0%)
Symptoms/poor quality of life from TSH suppression (118)	6 (5.1%)	10 (8.5%)	24 (20.3%)	62 (52.5%)	16 (13.6%)
Bone/CVS complications from TSH suppression (118)	5 (4.2%)	10 (8.5%)	27 (22.9%)	59 (50.0%)	17 (14.4%)

NA, not applicable; CVS, cardiovascular; TSH, thyroid stimulating hormone.

**Table 5:** The long term morbidity of the following are not well characterised

**Complication (number of responders)**	**Strongly agree**	**Disagree**	**Neutral/don’t know**	**Agree**	**Strongly agree**
Hypocalcaemia (118)	7 (5.9%)	32 (27.1%)	14 (11.9%)	59 (50.0%)	6 (5.1%)
Significant voice change (117)	12 (10.3%)	34 (29.1%)	12 (10.3%)	54 (46.2%)	5 (4.3%)
TSH suppression (116)	6 (5.2%)	19 (16.4%)	24 (20.7%)	57 (49.1%)	10 (8.6%)

NA, not applicable; CVS, cardiovascular; TSH, thyroid stimulating hormone.

## Discussion

To our knowledge this is the first survey evaluating perceptions of clinicians on treatment related morbidity of DTC.

Although almost half of the clinicians perceived morbidity associated with TSH suppression to be *‘rarely’* identified in clinic; only 2.5% and 4.0% had a formal protocol for detecting symptoms/poor quality of life from TSH suppression, and bone and cardiovascular complications respectively. It is unclear whether a lack of a formal protocol to detect these complications plays a role in the perception of morbidity. In addition, clinicians seeing higher volumes of patients with DTC perceived a higher frequency of long term hypocalcaemia, significant voice change and morbidity associated with TSH suppression (P < 0.05).

The lack of formal protocol to detect treatment related morbidity has also been confirmed by a recent survey of Japanese hospitals. They found most hospital did not perform BMD measurements in patients with TSH suppression for DTC. Less than 30% of hospitals routinely measure BMD in patients receiving TSH suppression for DTC. Of the hospitals that routinely measure BMD, measurements were taken yearly or twice yearly (44%), while 56% had no agreed interval for measurement [20].

Over 50% of clinicians perceived that the incidence of symptoms/poor quality of life from TSH suppression, and bone and cardiovascular complications of TSH suppression are not well defined. The picture was less clear with regards to temporary hypocalcaemia, long term hypocalcaemia and significant voice change: approximately equal numbers of clinicians ‘agree’ and ‘*disagree’* with the statement. Further analysis revealed that higher volume clinicians perceived that the incidence of temporary hypocalcaemia, long term hypocalcaemia and significant voice change are not well defined (P < 0.05).

Finally, over 50% of clinicians perceived that the long term morbidity of hypocalcaemia, significant voice change and TSH suppression are not well characterised. This in context of the overall perceptions over the clinical significance of treatment related morbidity (44.1% of clinicians perceived treatment related morbidity not to be a significant clinical problem) is of particular interest. One could postulate that it is difficult to ascertain the scale of a problem that is not well characterised or ill-defined.

One limitation of this survey was the inability to calculate the response rate due to significant overlaps between institutions contacted. The significance of bias due to non-response is therefore unclear. The potential impact of non-responders cannot be ignored as they may not have perceived treatment related morbidity in DTC to be a significant clinical problem. The survey only targeted surgeons and physicians who are members of specific organisations (TCF, BAETS and BTA), the findings may not be applicable to the entire population of thyroid surgeons and physicians.

The results reported are clinicians’ perceptions of treatment related morbidity; it may not mirror the reality or true scale of the problem. The results could have been skewed as surgeons represented the majority (67.8%) of the sample given; particularly, given that physicians seemed to perceive greater treatment related morbidity than surgeons. The difference between physicians and surgeons could be attributed to differences in current follow up protocols. The extent to which these groups of clinicians are involved in the follow up management of thyroid cancer patients is unclear and was not investigated in this survey. Another limitation of the survey was the relatively small proportion of clinicians seeing higher volumes of patients with DTC (28.8%); although it could be argued that this is more representative of current practice. This survey did not evaluate perceptions of morbidity associated with radioiodine ablation. Ascertainment of morbidity attributed to radioiodine ablation is important.

## Conclusion

There is a lack of formal protocols to detect complications associated with TSH suppression and most clinicians agreed that the incidence of complications associated with TSH suppression is not well defined. Over 50% of clinicians agreed that the long term morbidity of hypocalcaemia, significant voice change and TSH suppression are not well characterised. Physicians perceived treatment related morbidity to be a bigger problem than surgeons; this difference in perception could be related to differences in current follow up practices and differences in medical and surgical literature. A study of the prevalence and severity of treatment related morbidity and its impact on health of patients with DTC is warranted.

## Abbreviations

ATA: American Thyroid Association; BAETS: British Association of Endocrine and Thyroid Surgeons; BMD: Bone mineral density; BTA: British Thyroid Association; DTC: Differentiated thyroid cancer; SD: Standard deviation; TCF: Thyroid Cancer Forum; TSH: Thyroid stimulating hormone; UK: United Kingdom.

## Competing interests

The authors declare that they have no competing interests.

## Authors’ contributions

All authors were involved in conception, design and drafting of the manuscript. OE conducted the study and analysed the data. All authors read and approved the final manuscript.

## Authors’ information

Ovie Edafe, Medical Student.

Jon Wadsley, Consultant Oncologist.

Barney Harrison, Consultant Endocrine Surgeon.

Saba Balasubramanian, Clinical Senior Lecturer.
